# MENTAL HEALTH PROVIDERS’ PERSPECTIVES OF BALANCING RISK AND RESPONSE AND THE PANDEMIC’S IMPACT IN VHA NURSING HOMES

**DOI:** 10.1093/geroni/igae098.0079

**Published:** 2024-12-31

**Authors:** Kimberly Curyto, Kyle Page, Kristy Shoji, HyeRim Ryu, Tracey Washington, Michele Karel

**Affiliations:** Western New York VA Healthcare System, Batavia, New York, United States; Edward Hines, Jr. VA Hospital, Oak Park, Illinois, United States; Eastern Colorado Health Care System Rocky Mountain Regional VA, Aurora, Colorado, United States; Edward Hines, Jr. VA Hospital, Oak Park, Illinois, United States; Captain James A. Lovell Federal Health Care Center, North Chicago, Illinois, United States; VA Central Office, Washington, District of Columbia, United States

## Abstract

The COVID-19 pandemic forced a reconceptualization of how mental health services were offered to older adults in nursing homes, affecting residents and staff, as each experienced stringent and evolving infection control precautions. An anonymous national survey of Veterans Health Administration Community Living Center (CLC) mental health professionals in October 2022 included questions about the impact of the pandemic on CLC residents and healthcare teams. Respondents (N=107) included psychologists (82%) and psychiatric providers (18%). Sixty-six percent of respondents worked in the CLC before the pandemic and reported providing mostly in-person clinical care (97%). In comparison, across the entire sample two-years later, 72% reported providing in-person clinical care, 23% a mix of in-person and virtual care, and 5% entirely virtual care. An expert panel coded responses to the question, please describe your work with Veterans and teams in the CLC during the pandemic, in rotating groups of two to identify qualitative themes, and reviewed as a group to achieve consensus. Several themes emerged: 1) varied and changing methods/modes of work, 2) continued psychotherapy and assessment services, 3) increased education and support for the team, 4) declines in resident engagement, mental health, and cognition, and 5) negative impact on providers/the team (e.g., barriers to teamwork and person-centered care). Results underscore the impact the pandemic/precautions had on residents’ and teams’ well-being, as well as the adaptability of mental health providers, and may inform ongoing and future efforts to balance risk and support person-centered care.

